# Growth factor dependency in mammary organoids regulates ductal morphogenesis during organ regeneration

**DOI:** 10.1038/s41598-022-11224-6

**Published:** 2022-05-03

**Authors:** Sounak Sahu, Mary E. Albaugh, Betty K. Martin, Nimit L. Patel, Lisa Riffle, Susan Mackem, Joseph D. Kalen, Shyam K. Sharan

**Affiliations:** 1 Mouse Cancer Genetics Program, Centre for Cancer Research, National Cancer Institute, Bldg- 560, Room 32-33, 1050 Boyles Street, Frederick, MD 21702 USA; 2 Leidos Biomedical Sciences, Inc., Frederick National Laboratory for Cancer Research, Frederick, MD 21702 USA; 3 Small Animal Imaging Program, Frederick National Laboratory for Cancer Research, Frederick, MD 21702 USA; 4 Cancer and Developmental Biology Laboratory, Centre for Cancer Research, National Cancer Institute, Frederick, MD 21702 USA; 5 Centre for Advanced Preclinical Research, National Cancer Institute, Bldg- 560, Room 32-33, 1050 Boyles Street, Frederick, MD 21702 USA

**Keywords:** Biological techniques, Developmental biology

## Abstract

Signaling pathways play an important role in cell fate determination in stem cells and regulate a plethora of developmental programs, the dysregulation of which can lead to human diseases. Growth factors (GFs) regulating these signaling pathways therefore play a major role in the plasticity of adult stem cells and modulate cellular differentiation and tissue repair outcomes. We consider murine mammary organoid generation from self-organizing adult stem cells as a tool to understand the role of GFs in organ development and tissue regeneration. The astounding capacity of mammary organoids to regenerate a gland in vivo after transplantation makes it a convenient model to study organ regeneration. We show organoids grown in suspension with minimal concentration of Matrigel and in the presence of a cocktail of GFs regulating EGF and FGF signaling can recapitulate key epithelial layers of adult mammary gland. We establish a toolkit utilizing in vivo whole animal imaging and ultrasound imaging combined with ex vivo approaches including tissue clearing and confocal imaging to study organ regeneration and ductal morphogenesis. Although the organoid structures were severely impaired in vitro when cultured in the presence of individual GFs, ex vivo imaging revealed ductal branching after transplantation albeit with significantly reduced number of terminal end buds. We anticipate these imaging modalities will open novel avenues to study mammary gland morphogenesis in vivo and can be beneficial for monitoring mammary tumor progression in pre-clinical and clinical settings.

## Introduction

The dynamics and plasticity of adult stem cells (ASCs) play a major role in postnatal organogenesis and cellular interaction during organ development. The remarkable capacity of mouse mammary stem cells (MaSCs) to regenerate mammary ductal tree upon transplantation has opened new avenues to visualize development and tissue repair in vivo^1,2^. The mammary gland undergoes extensive remodeling during puberty and pregnancy, and understanding how MaSCs contribute to post-natal organogenesis has immense implications for its oncogenic transformation^3^, as well as relevance for regenerative biology. Rapidly expanding toolboxes have uncovered the genetic heterogeneity and lineage specification of ASCs in mammary gland^4,5^, but imaging tools to accurately study the role of ASCs in organ regeneration and tissue morphogenesis are just beginning to be unveiled.

The self-organizing property of ASCs to form 3D organ-like structures or organoids, has revolutionized our ability to model and understand organogenesis and disease progression^6–8^. Organoids can be generated from multiple adult tissue types and from tumors that can be propagated as well as cryopreserved^9–12^. Compared to other tissue-specific organoids, the capacity of mammary organoids to regenerate an entire organ system with distinct cellular compartment makes it a convenient model to understand development and regeneration in vivo. Several approaches have been taken to generate mammary organoids from both human and mouse mammary glands and from healthy and cancer tissues^12–19^. Multiple studies have shown the role of tissue geometry, underlying basement matrix (like Matrigel, Collagen) and growth factors (GFs) that can affect differentiation, development and lineage specification in mammary organoids and mammospheres, and invasion in tumor organoids^16,17,20–22^ However, the role of individual growth factors (GFs) and cytokines in 3D in vitro culture and its impact on organ transplantation in vivo is still understudied. GFs promote the signal transduction and dysregulation of which often leads to cell transformation and tumorigenesis. The interaction between epithelial cells and the underlying mesenchyme during embryogenesis instructs the differentiation trajectory of MaSCs and their progenitors^23,24^. Hence the signals from the niche play an important role in regulating MaSCs properties^25^. Several studies have reported the role of EGF ligands and FGF signaling to maintain organoids in culture^13,14,26,27^, but their effect on mammary organoid maintenance and its impact in organ regeneration in vivo is limited.

Understanding tissue regeneration in vivo relies on performing serial histological sections or intravital imaging that are immensely time consuming and labor intensive. On the contrary, the Carmine-Alum staining of the ductal trees of the mammary gland has been a gold-standard tool to examine the ability of mammary epithelial tissues to reconstitute a whole organ. Mammary ductal tree regeneration from inaccurate fat pad clearing will also be stained using Carmine Alum, thereby leading to erroneous data in mammary tree reconstitution. Hence, relying on Carmine-Alum staining to determine the accuracy of reconstitution from donor epithelium could lead to false-positives. To overcome these shortcomings, here, we utilized multi-scale imaging techniques using in vivo whole animal fluorescence and high-resolution confocal microscopy with tissue clearing strategies to understand organ regeneration. tdTomato (tandem dimer Tomato) is an engineered genetic fusion of two copies of dTomato that gives exceptional brightness and photostability^28,29^, hence we selected it as an ideal fluorescent reporter for in vivo imaging. High-throughput in vivo imaging is often limited to the size and thickness of the biological sample leading to decreased signal intensity. The advent of intravital imaging and high resolution microscopy of large specimens coupled with tissue-clearing strategies has enhanced our understanding on organ development by overcoming tissue thickness^30–33^. While intravital microscopy provides better resolution and precision, it requires advanced microsurgical and image analysis expertise, and can be often time-consuming. Automated imaging systems have also been developed using tools that are able to spatially resolve organ structures with increased depth and reduced acquisition time^34^. While a variety of tissue clearing techniques have been developed such as 3DISCO, CLARITY, CUBIC, and SHANEL, that facilitate high-throughput imaging of thick specimens, each has its own limitations^35^. The fructose-glycerol (FG) based tissue clearing approach is non-toxic and has been previously used for high-resolution imaging of several types of organoids and tumors^36,37^. We extend the potential of this approach to facilitate in situ 3D imaging with more clarity and rapidity to investigate how GFs during 3D in vitro organoid culture can impact organ regeneration in vivo*.*

## Results

### Combination of EGF and FGF signaling is necessary for mammary organoid maintenance in vitro

**Figure 1:**
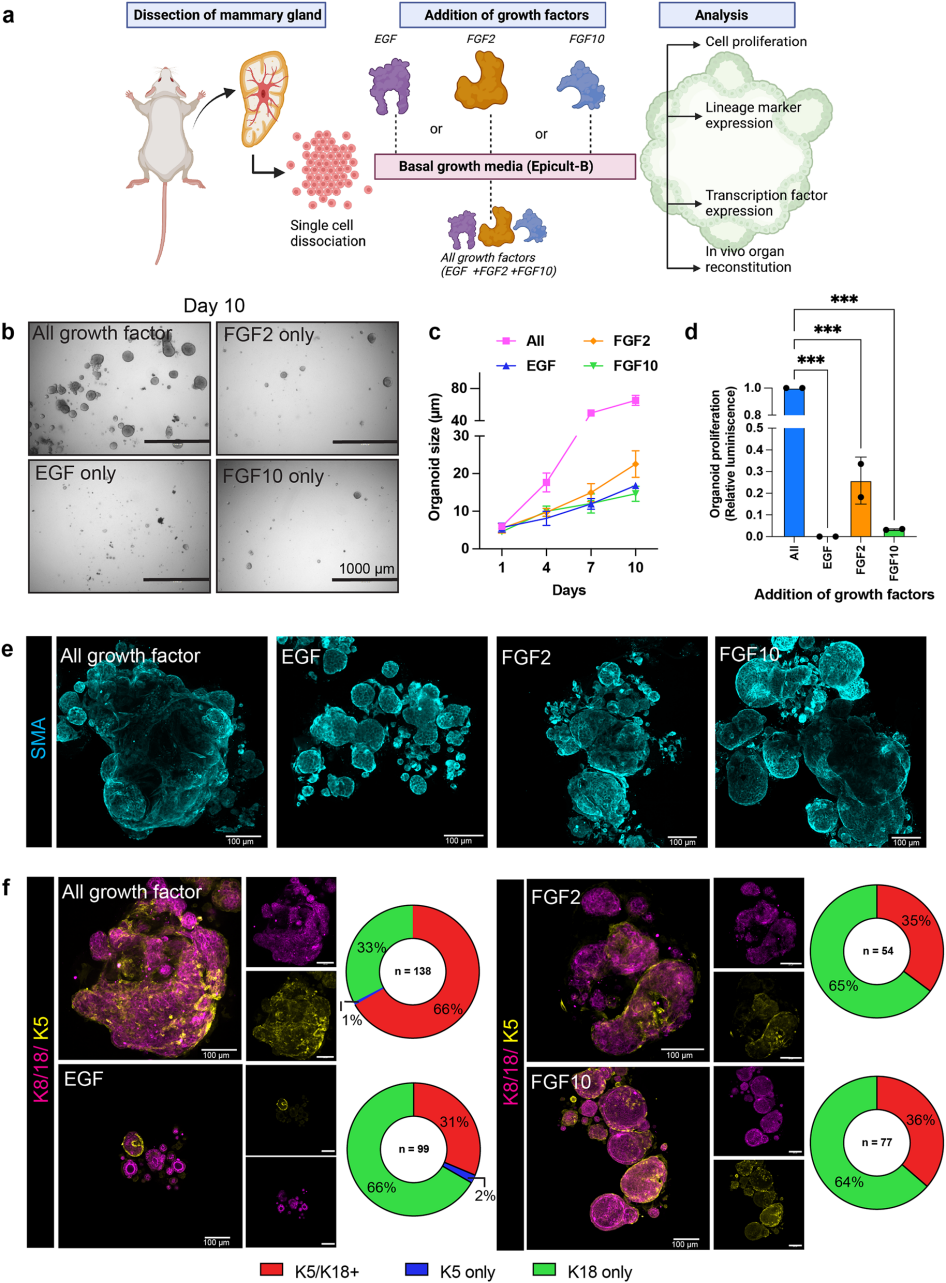
Combinatorial signaling of EGF, FGF2 and FGF10 are required for mammary organoid maintenance. (**a**) Experimental schematic showing mammary glands were surgically dissected and dissociated into single cells. Organoids were grown from single cells and cultured in 3D in the presence of EGF or FGF2 or FGF10 recombinant proteins alone or in a cocktail of all growth factors. The mammary organoids were further analyzed for cell proliferation, expression of mammary lineage markers and transcription factors, and the ability to undergo in vivo organ reconstitution. (**b**) Representative images showing the size of organoids cultured in a combination of growth factors [All] as compared to the addition of individual growth factors in the basal organoid media at day 10 (Scale bar = 1000 µm). (**c**) Quantification showing the growth of organoids over a time course starting at day 1 to day 10 (n > 50 organoids were counted per time-point per condition). (**d**) Organoid proliferation is maintained when cultured in a cocktail of growth factors including EGF, FGF2 and FGF10. Results are expressed as mean ± SD where each dot represents a biological replicate (n = 2) and values were averaged and normalized from three technical replicates. One way ANOVA multiple comparison test was used for computing statistical significance (***p < 0.001). (**e**) Representative images showing expression of myoepithelial cell markers—Smooth Muscle Actin (SMA, Cyan) in organoids grown under different growth factor condition (Scale bar = 100 µm). (**f**) Immunofluorescent staining showing the maintenance of Cytokeratin 8/18 (Magenta, Luminal epithelial cell markers) and Cytokeratin 5 (Yellow, basal epithelial cell marker) (Scale bar = 100 µm). The pie chart represents the proportion of K8/18^+^K5^+^, K8/18^+^K5^−^ and K8/18^−^K5^+^ organoids present in each condition. Statistical significance is calculated by Fisher’s Exact test (Two-tailed *p* value < 0.0001 compared to “All growth factors” condition).

Self-organization of mammary stem cells into 3D organoids provides a robust in vitro system to study the regulatory interplay of growth factors (GFs) in adult stem cell maintenance. Studies using 3D cell culture system from different organoid types have demonstrated the role of EGF and FGF signaling in stem cell survival, differentiation, epithelial morphogenesis and long-term expansion of organoids in vitro^27,38^. Here we investigate the role of EGF and FGF signaling factors in mammary organoid maintenance and proliferation in vitro and their impact on organ regeneration in vivo (Fig. 1a). The 4th inguinal mouse mammary glands were dissected out and chopped into small pieces and digested with collagenase-hyaluronidase. Mammary organoids are routinely cultured in 80–100% Matrigel that provides support to develop complex 3D structures^16^. While Matrigel has been widely used for decades in cell culture, recent studies report variability in cell culture experiments owing to the ill-defined composition and batch-variability^39,40^. Here we develop a suspension culture method with minimal Matrigel to generate 3D mammary organoids. Freshly isolated mammary epithelial cells were cultured in basal Epicult-B medium (murine grade) containing insulin, Heparin and Hydrocortisone and a fixed concentration of different GFs that are previously identified to regulate mammary gland development^13,14,20,27^. We took an individualistic approach to investigate the role of GFs in organoid culture and observed that the growth and size of the organoids over a time course of 10 days is significantly impaired when cultured with EGF or FGF2 or FGF10 alone as compared to the addition of a cocktail of the GFs in basal media (Fig. 1b, c and Supp. Fig. 1a, b). The proliferation of the organoids was also significantly enhanced when grown in the cocktail of GFs (Fig. 1d). We next compared the efficiency of our minimal Matrigel suspension culture method to previously established complete Matrigel embedding methods^13,14^. The ability to form organoids in 2% Matrigel is equally efficient when compared to Matrigel embedding methods (Supp. Fig. 1c), with similar distribution of branched to compact organoids formed in the presence of different growth factors (Supp. Fig. 1d–g). We next examined the organization of the epithelial architecture in organoids that were grown in the presence of GFs.

The basal and luminal epithelial cells differentially express cytokeratin proteins that characteristically define mammary epithelium. The basal epithelium consisting of myoepithelial cells marked by cytokeratin markers (K5, K14) and Smooth muscle Actin (SMA) generate the outer layer of the gland. The luminal epithelial cells marked by cytokeratin (K8/K18) forms ducts and secretory alveoli and also consist of hormone-sensing cells expressing Estrogen receptor (ER^+^) and Progesterone receptor (PR^+^)^41^. Despite the smaller size of the organoids, myoepithelial cells marked by SMA are present in all the experimental conditions (Fig. 1e). The luminal K8/18^+^ cells and the basal K14^+^ cells were maintained in organoids grown in the presence of a single or cocktail of GFs, thereby recapitulating the epithelial cell types present in adult mammary gland in vivo (Supp. Fig. 2a). However, the proportion of organoids lined by inner luminal K8/18^+^ cells and surrounded by basal layer of K5^+^ cells are higher when organoids are cultured in a cocktail of GFs containing EGF, FGF2 and FGF10 (Fig. 1f).

### Growth factor dependence on the maintenance of mammary stem cell-specific transcription factor expression

**Figure 2:**
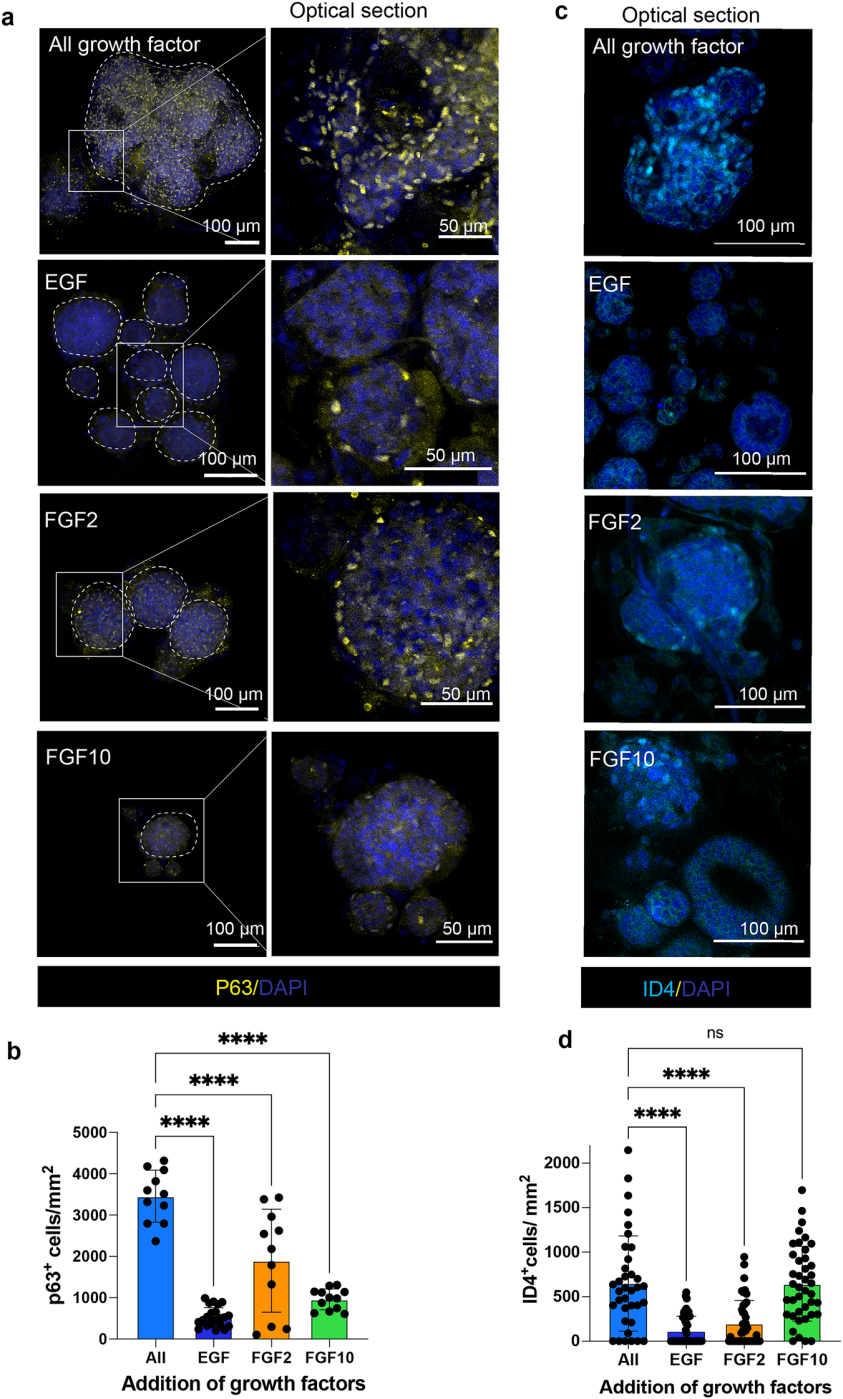
Mammary stem cell-specific transcription factors are expressed in organoids cultured in a cocktail of growth factors. (**a**) Representative images showing the expression of transcription factor p63, mostly expressed in basal epithelial cells in each condition (Scale bar = 100 µm). (**b**) Quantification showing the significant increase in p63 + cells in individual organoids cultured in a cocktail of growth factors as compared to EGF, FGF2, or FGF10 alone. Each dot represents total cells/mm^2^ in each organoid. Results are expressed as mean ± SD and one-way ANOVA for multiple comparison test was used to measure statistical significance. (n = 11–23 organoids per condition). (**c**) Representative images showing the expression of transcriptional factor ID4 in mammary organoids cultured in individual growth factors (Scale bar = 100 µm). (**d**) Quantification showing the significant increase in ID4 + cells in individual organoids cultured in a cocktail of growth factors. Results are expressed as mean ± SD and one-way ANOVA for multiple comparison test was used to measure statistical significance. (n = 36–44 organoids per condition). Each dot represents total cells/mm^2^ in each organoid.

GFs or cytokines play a pivotal role in epithelial cell maintenance and transduce their effects on gene transcription through recognition of specific receptors and activation of transcription factors (TFs). Mammary epithelial cells express several TFs that mediate lineage differentiation and mammary stem cell maintenance. Some of the key TFs involved in mammary morphogenesis includes P63, SOX9, ID4, STAT5 that are distributed throughout different mammary epithelial lineage. P63 is selectively expressed only in basal epithelial cells and also required for embryonic mammary development^42,43^. We observed P63^+^ cells in organoids grown in the cocktail of GFs and a stark decrease when grown in EGF or FGF2 or FGF10 alone (Fig. 2a, b). This is concomitant with the decrease in basal epithelial lineage, marked by K5 (Fig. 1e). Another TF expressed in basal epithelial lineage, Inhibitor of differentiation 4 (ID4), is a key regulator of mammary stem cells and also prevents luminal and myoepithelial cell differentiation^44–47^. We observed a significant reduction in ID4^+^ cells in organoids grown in EGF and FGF2 alone as compared to the cocktail of GFs (Fig. 2c, d). Apart from epithelial cells, mammary stem cells differentiate to give rise to hormone-responsive cell types. The mammary epithelial cells can express receptors responsive to estrogen (ER) and progesterone (PR) hormone signaling, which represents two independent lineages that are maintained by stem/progenitor cells^48,49^. Despite the skewed proportion of luminal and basal epithelial lineages, the organoids can maintain the hormone responsive cells, except in the organoids grown in EGF alone where the ER^+^ and PR^+^ cells are significantly reduced (Supp. Fig. 2b, c). Our data suggests that minimal Matrigel concentration can also form mammary organoids in suspension with distinct epithelial cell layer that resemble very closely with the tissue architecture of an adult mammary gland.

We next investigated if a combination of GFs can also maintain organoids size and epithelial lineage markers. Single cells isolated from adult mammary gland were cultured in 2% Matrigel suspension culture and in the presence of two GFs (EGF and FGF2, EGF and FGF10, FGF2 and FGF10) (Supp. Fig. 2d). We have observed organoids of comparable size when a single GF is not added, except in the absence of FGF2, where organoid size and proliferation is significantly impaired (Supp. Fig. 2e–g). We also found organoids grown in absence of FGF2 has reduced organoid size over a time course of 10 days as compared to other GF combination and also when compared to the cocktail of all the GFs (Supp. Fig. 2h). Despite small size in organoids, basal and luminal epithelial markers are present in organoids cultured in different GF combination (Supp. Fig. 2i). Our data suggest, while there is a redundancy in the effect of GFs, FGF2 plays a critical role in mammary organoid maintenance.

### Imaging modalities allows visualization of organ development and tissue repair in vivo

**Figure 3:**
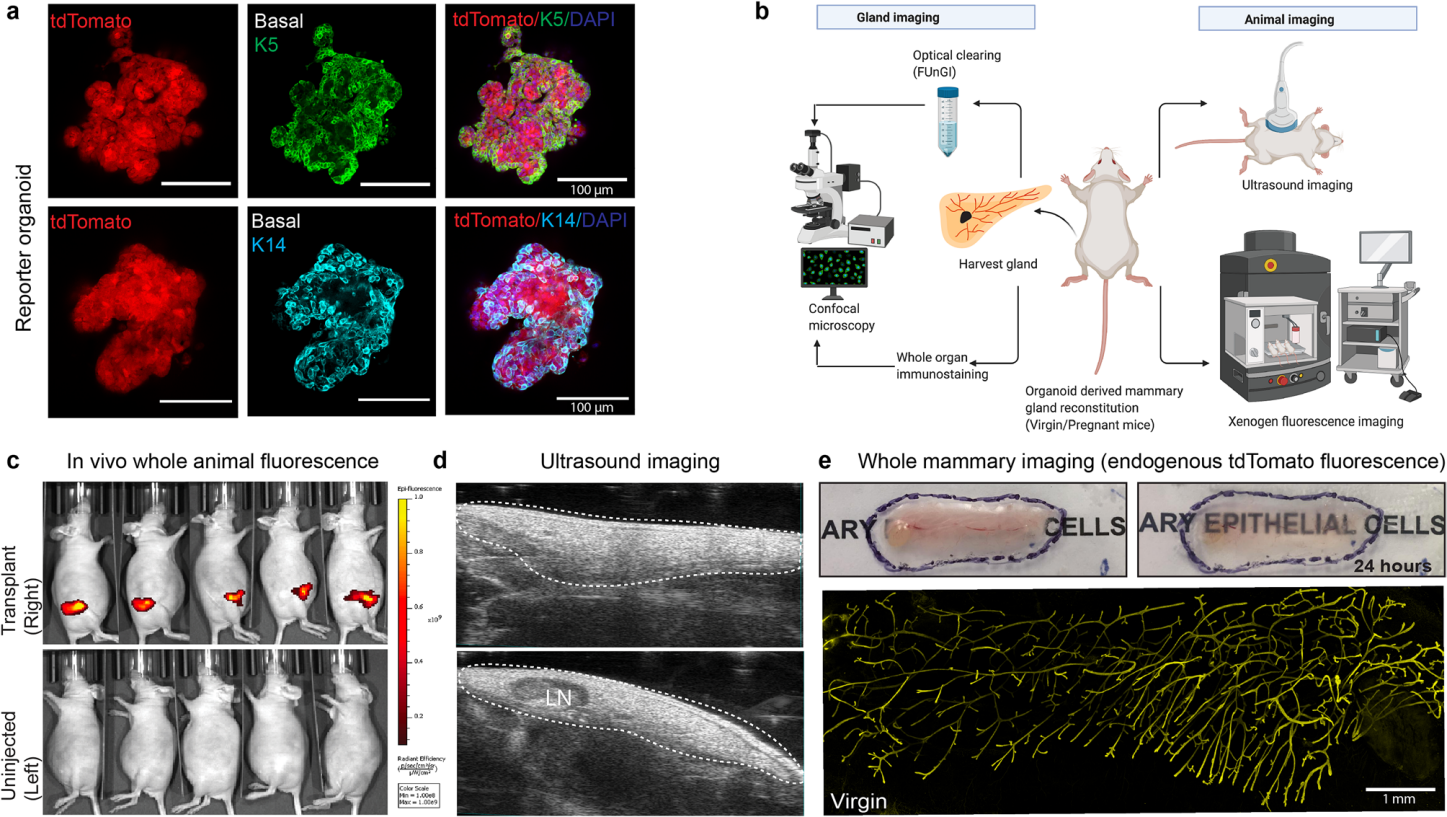
Imaging modality to whole animal in vivo fluorescence. (**a**) Immunofluorescent staining showing the expression of basal epithelial marker (K5 and K14) in mammary organoids generated from Rosa26 tdTomato mammary gland. The Red fluorescence is tdTomato endogenous fluorescence (Scale bar = 100 µm). (**b**) Schematic showing imaging modalities to whole gland imaging and whole animal imaging. Animals were anaesthetized after 4 weeks post transplantation and imaged for tdTomato fluorescence at the left side (site for transplantation) and also checked for mammary fatpad imaging through ultrasound imaging. The mammary glands were dissected out and checked for the endogenous tdTomato fluorescence to visualize ductal trees, and also performed whole-gland immunostaining to check tdTomato-derived mammary epithelial cells. (**c**) In vivo whole animal fluorescence (Xenogen IVIS fluorescence imager) reveals tdTomato expression at the site of transplantation. The right side was used as control (n = 5 pregnant mice). The Radiant efficiency scale in the whole animal fluorescence is expressed as (p/sec/cm^2^/sr)/(µW/cm^2^). Color scale: Min = 1.00e^8^ and Max = 1.00e^9^. (**d**) Ultrasound imaging reveals the formation of mammary fat pad and complete absence of the lymph node (LN) confirming efficient mammary fat pad clearing and the ability of mammary organoids to reconstitute mammary gland (n = 5 pregnant mice). (**e**) Tissue clearing enables 3D confocal imaging to visualize mammary ductal tree formation. The mammary glands were surgically dissected after 4 weeks post transplantation (virgin) and incubated in Fructose-Glycerol clearing Solution (FGCS) for 24 h, making the whole tissue translucent for efficient imaging. Tissue clearing can also be performed for 3–7 days (Pregnant mice) depending on the stage of mammary gland development. Confocal microscopy of the whole gland was subsequently imaged to check for the presence of ductal trees, and the endogenous tdTomato fluorescence confirms organoids are able to regenerate mammary gland ductal tree in vivo (Scale bar = 1 mm).

Organoids have been previously used to study branching morphogenesis in vitro, leading to the identification of GFs and surrounding microenvironmental cues that regulate this process^50^. Here we present imaging modalities extending from established imaging toolbox to understand how organoids grown under different GFs contribute to ductal regeneration in vivo. We took advantage of native fluorescence that can be maintained for a long time with minimal loss of fluorescence during imaging. We generated mammary organoids from Rosa26-tdTomato-active mice that expresses strong red fluorescence ubiquitously in all organs. Using the conditions optimized previously (Fig. 1a), we isolated the 4th inguinal mammary glands from both sides and the dissociated single cells were cultured in vitro for 2 weeks under 3D conditions using a cocktail of GFs to generate mammary organoids. We confirmed the tdTomato expression in the organoids that also expresses basal epithelial lineage (marked by K5 and K14) (Fig. 3a). The Rosa-tdTomato organoids were partially dissociated, and 10^4^ cells mixed in organoid media containing 30% Matrigel were transplanted into the cleared mammary fat pad of a 3-week-old athymic nude mice (right side of the 4th inguinal mammary gland). We next optimized imaging modalities to visualize organ regeneration by performing multi-spectral imaging followed by gland imaging with endogenous tdTomato reporter expression (Fig. 3b). Multispectral fluorescence was excited at 430 nm to obtain autofluorescence spectra) and at 535 nm (to obtain tdTomato spectra); Em 580, 600, 620, 640, 660, 680 nm using a Xenogen IVIS Spectrum imager (PerkinElmer, Waltham, MA) (Supp. Fig. 3a). The spectral unmixing (to correct for autofluorescence) was performed and whole-animal fluorescence imaging revealed a significant red fluorescence at the transplanted region as compared to the un-injected (left side) control mammary gland (Fig. 3c and Supp. Fig. 3b, c). After fluorescence imaging, the mammary fat pad was ultrasound imaged in B-mode (Vevo 2100, VisualSonics, Toronto, Canada) using a MS-550S 40 MHz transducer without waking them up. The ultrasound 3D B-mode revealed the presence of thick tissue at the right side and absence of lymph node confirming successful fat pad clearing and growth of the mammary epithelium (Fig. 3d and Supp. Fig. 3d). However, the thick fat pad tissue does not necessarily reveal the formation of ductal tree after transplantation. To further confirm that the organoids can generate mammary ducts and are not just clumped at the site of injection, we dissected out the mammary glands from the injected site and checked for the expression of tdTomato in the fat pad using ex-vivo confocal imaging.

The high scattering of lights from the large and fatty structure of mouse mammary gland reduces the signal intensity, thereby making it difficult to perform optical imaging. We utilized Fructose and Glycerol Clearing Solution (FGCS) to visualize the mammary gland ductal tree reconstitution. The 4th mammary gland was removed and incubated in FGCS for 3 days and then imaged to visualize the endogenous tdTomato expressing mammary ducts. FGCS is a non-toxic tissue clearing approach allowing faster imaging with minimal light scattering and organ translucency is observed within 24 h of incubation (Fig. 3e). However, depending on the abundance of the surrounding fatty tissue and stage of mammary gland development, the time for tissue clearing was optimized for virgin gland (24 h) and for pregnant tissue (3–7 days due to thick and fatty tissues). FGCS-based tissue clearing allowed us to image bright tdTomato fluorescence which further confirms that mammary ductal tree is generated from tdTomato expressing organoids.

### Transplanted organoids can regenerate functional organ

**Figure 4:**
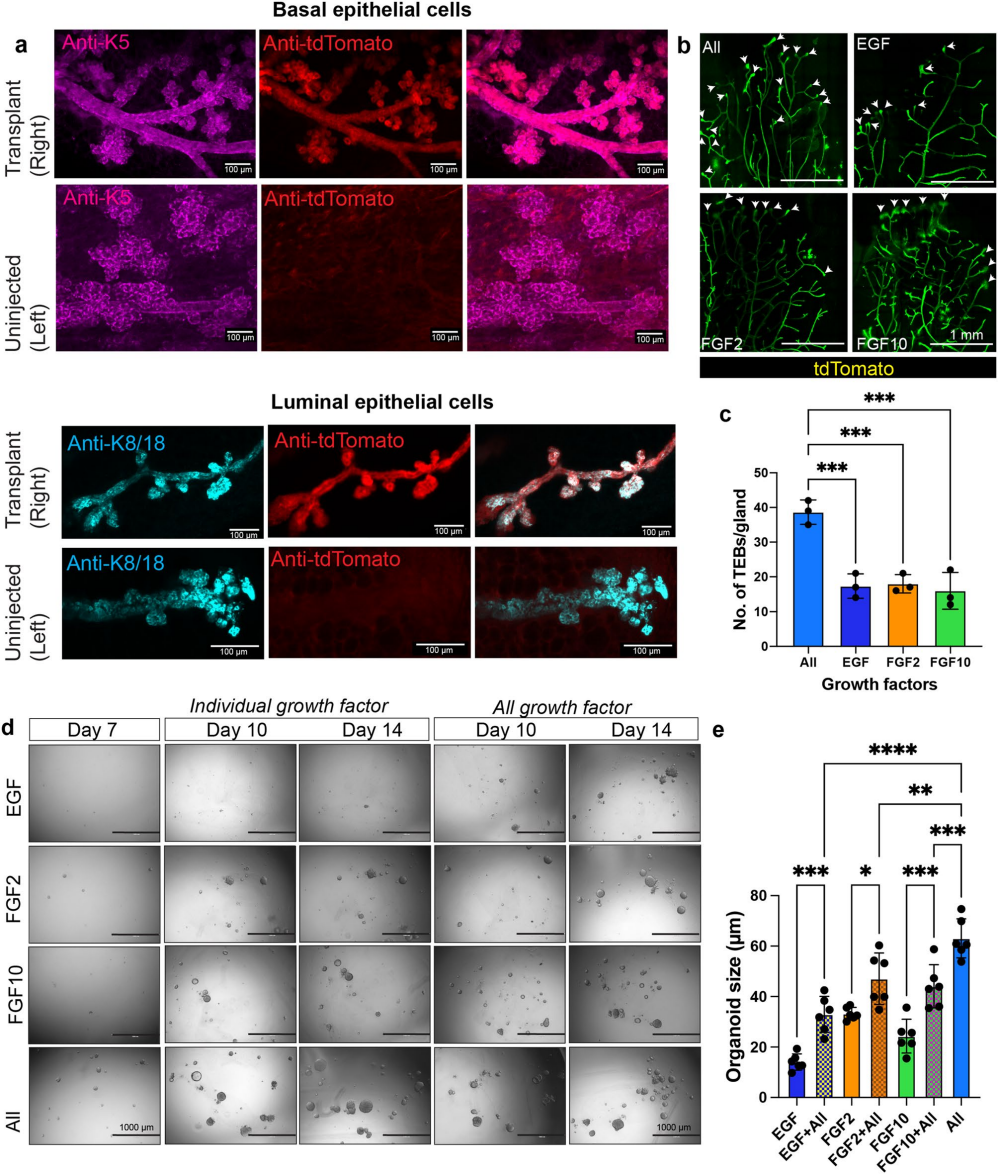
Effect of growth factors on organ regeneration. (**a**) Whole gland immunostaining reveals the development of K5^+^ basal mammary epithelial cells (Magenta) and K8/18^+^ luminal epithelial cells (Cyan) in pregnant mice generated after transplantation of tdTomato + mammary organoids in the left side. The un-injected control showed only the presence of K5 basal epithelial cells and complete absence of tdTomato staining (Scale bar = 100 µm). Anti-DsRed was used to detect td-Tomato which is a derived mutant of DsRed. (**b**) Whole gland imaging for endogenous tdTomato fluorescence to check the effect of growth factors on their ability to generate mammary repopulating units (MRUs). Organoids grown in EGF, FGF2, FGF10 or in a combination of growth factors were partially digested and 10^4^ cells were transplanted into the left mammary fat pad after surgically removing the endogenous mammary epithelium. Arrow heads mark the terminal end buds (TEBs). (**c**) Quantification showing the significant reduction in the formation of TEBs after transplantation of organoids grown in different mammary specific growth factors. (scale bar = 1 mm, n = 3 mice per condition). (**d**) Representative brightfield images of organoids grown in the presence of single GFs for 7 days. “All GFs” containing EGF, FGF2, FGF10 were added to the single GF-grown organoids from 7th to 14th day. A counter panel of organoids were grown in the presence of “individual GFs” as a control. Organoids grown in the cocktail of GFs from day 0 were also used a control. Scale bar = 1000 µm. (**e**) Organoid size were measured from individual organoids and each dot represents average size of organoids from each replicate (n = 2 biological replicates containing 3 technical replicates each). One way ANOVA was used to measure statistical significance (*p < 0.05, **p < 0.01, ***p < 0.001, ****p < 0.0001).

We next investigated if the transplanted organoids could regenerate the distinct epithelial layers. We performed whole-mount immunostaining on the 4th mammary gland from both the injected and un-injected sides, and stained with anti-K5 (basal epithelial cell marker) and anti-DsRed (to detect td-Tomato which is a derived mutant of DsRed^28^). The glands were incubated in FGCS reagent for tissue clearing post immunostaining followed by 3D confocal imaging, which revealed the presence of basal epithelial cells marked by K5 along the mammary ducts that also show strong tdTomato fluorescence. Similarly, the dissected glands also showed the presence of luminal epithelial cells marked by K8/18 along with strong tdTomato expression (Fig. 4a). In contrast, the un-injected control side also showed the presence of K5^+^ and K8/18^+^ staining along the mammary ducts but there was complete absence of tdTomato fluorescence. This further confirms that the transplantation of organoids that are cultured in the cocktail of GFs can reconstitute a functional mammary gland with bilayered epithelial architecture. The mammary ducts undergo massive remodeling during pregnancy and are associated with increased proliferation and alveolar morphogenesis for milk secretion. We observed the ductal trees that are regenerated from organoids can respond to hormones during the pregnancy cycle and proliferate to form lobulo-alveloar units (Supp. Fig. 4a). Taken together, our results support FGCS-tissue clearing approach is a robust and powerful tool to visualize cellular architecture with strong fluorescence signal intensity.

GFs induce branching morphogenesis in mammary organoids cultured in Collagen I matrix or Matrigel in vitro^16,17,26^ but how does this translates to organ regeneration in vivo is unknown*.* Utilizing the imaging toolbox that we have previously established*,* we next investigated how organoids grown in different GFs are able to regenerate mammary ductal tree. Mammary organoids were generated from Rosa26-tdtomato expressing mammary gland and individual or a cocktail of GFs were added (referred to “All” as described in Fig. 1a). The organoids grown under different GFs conditions were transplanted to cleared mammary fat pad (4th inguinal) of 3-week-old immunocompromised mice and the mammary glands were dissected at 4 weeks post transplantation for visualization of organ regeneration. Despite the smaller size of the organoids when grown in the presence of individual growth factors (Fig. 1b, c), they have the ability to repopulate a cleared fat pad with mammary ductal branching (Supp. Fig. 4b), supporting previous in vitro results^17^. However, the mammary repopulating units developed from organoids that were grown in a cocktail of growth factors were able to form significantly high number of terminal end buds (TEBs) and increased branch points compared to the organoids cultured in the presence of EGF or FGF2 or FGF10 alone (Fig. 4b, c, Supp. Fig. 4b–d). We have observed considerable branching in mammary glands regenerated from the organoids grown in vitro in the presence of different GFs. The GFs used in our in vitro experiments will also be present during in vivo ductal morphogenesis. We next focused on studying the role of these GFs that can impact ductal branching post transplantation. Organoids derived from mammary epithelial cells were grown for 7 days in the presence of single GFs and then substituted with media containing EGF + FGF2 + FGF10 GFs and cultured for another 7 days and checked for organoid maintenance. (Supp. Fig. 4e). We observed a significant increase in organoid size over a time course from 7th to 14th day when single GFs were replaced with a cocktail of GFs in all the conditions as compared to growing them in single GFs (Fig. 4d, e and Supp. Fig. 4f). Some of the organoids can also maintain the luminal epithelial cells along with an underlying basal epithelial layer. (Supp. Fig. 4g). While there is a significant increase in organoid size when cocktail of GFs was added to single GF-grown organoids, it is partially rescued and does not increase to the same extent as to the organoids that are grown in presence of all GFs from the beginning (Fig. 4e). Overall, our data suggests, there is an impact on ductal branching from GFs present in the mammary fat pad microenvironment. The reduced TEBs observed in mammary transplant is due to the growth factor dependency of organoids grown in vitro*.*

## Discussion

GFs regulating EGF and FGF signaling play major roles in the mammary epithelium during embryogenesis, post-natal development and has immense implications in breast cancer progression^20,51–53^. Technical differences exist in maintaining mammary organoids but are mostly grown with an underlying basement matrix to reproduce the near physiological state of an organ. Different concentrations of Matrigel or defined mixtures of Matrigel and collagen have been classically used as a matrix to culture and propagate mammary organoids^16,17,50,54^. Although, Matrigel has been utilized for decades it often shows variable protein concentrations and batch-to-batch variability. Matrigel being an animal-derived product impose bottleneck to bulk production and its ill-defined composition inspire the development of synthetic scaffold alternatives^40^ or generate organoids as suspension culture^55^. In this study we developed a suspension culture method using minimal concentration of Matrigel that provides the extracellular support to generate 3D organoids. We found that a cocktail of GFs containing EGF, FGF2 and FGF10 along with basal Epicult-B media containing Heparin, Hydrocortisone, and Insulin can maintain cell proliferation and size of the 3D organoids in vitro. These organoids also contain distinct luminal and basal epithelial lineages marked by cytokeratin markers that recapitulate an adult mammary gland. We have also shown that TFs (p63 and ID4) required for mammary stem cell maintenance are expressed in organoids cultured in the cocktail of GFs. Organoids grown in the presence of EGF alone are severely impaired in their size and proliferative ability. Organoids generated from human mammary tissue samples revealed that removal of EGF led to an increase in mature luminal cells but decrease in basal cells^56^. Our current work using bulk mammary epithelial cells showed organoids can maintain distinct basal and luminal epithelial cells in the absence of only EGF but limited to comment if organoids generated are entirely luminal or basal-derived. Future work on isolating individual cell population in mouse mammary gland will reveal how GFs can impact maintenance of specific cell types. We found branching of organoids in our suspension culture and FGF2 alone can lead to branching in organoids as previously reported^20^, but the frequency increases when added in combination of all GFs. To better understand the effect of single GFs, we investigated the effect of the combination of two GFs and found FGF2 to play a critical role in maintaining mammary organoid size and proliferation. Despite the smaller size, the organoids can maintain their epithelial lineage cell types, suggesting combination of EGF and FGF signaling is required to culture and maintain organoids. FGF signaling has previously been shown to play pleiotropic roles in mammary morphogenesis and also during lactation^20,26^ and fetal mammary stem cells grown in suspension also led to higher sphere formation in the presence of EGF and FGF together as compared to single GF^57^.

We next investigated if the organoids grown in the presence of different GFs can regenerate the mammary gland. The mammary fat pad transplantation assay is a gold-standard technique to study mammary epithelial organogenesis and is visualized using Carmine-Alum whole-mount staining or bioluminescence imaging (BLI)^58^. Carmine alum staining does not convincingly capture the donor epithelium and any remaining host mammary epithelium will interfere in visualizing mammary ductal tree formation. In contrast, BLI provides valuable insights to key biological concepts with immense application in vivo for non-invasive monitoring of biological phenomena and has been used to track the contribution of luciferase promoter driven expression in cells in vivo. However, limitations include solubility of the substrate, lower bioluminescence signal in thick tissues and variable substrate intake in bigger tumors leading to erroneous data^59^. It also requires an invasive and toxic luciferin drug to be administered. To this end the ultrasound imaging is a rapid and non-invasive method to differentiate between soft tissue and cysts/mass that can be a useful approach to correlate findings derived from whole animal imaging and ex vivo confocal imaging. Considering the thickness of the mammary gland owing to the presence of stromal fibroblast and adipocytes, makes it difficult to perform whole-tissue confocal imaging. Several optical clearing methods (like iDISCO, CUBIC, CLARITY) have been developed to visualize thick tissues and organoids but are often laborious and time consuming compared to Fructose and glycerol-based clearing agents (FGCS or FUnGI)^36^. In this study, we developed fluorescence-based imaging modalities to visualize organ regeneration by utilizing a brighter fluorescent reporter and robust tissue clearing approach. We used td-Tomato fluorescence which is as photostable as mCherry and six times brighter than eGFP, thus allowing deeper penetration in thick tissues^28,29^. FGCS tissue clearing allowed faster imaging of mammary glands with maximum accuracy to confirm organoids can regenerate mammary ductal trees. We have shown ductal trees generated from tdTomato expressing organoids that are grown in the presence of all GFs can form distinct bulb-shaped terminal end buds (TEBs). Tissue clearing followed by whole-gland confocal imaging also revealed that organoids grown in cocktail of GFs can regenerate a significantly higher number of TEBs. The leading-edge bulbs of these TEBs consists of highly proliferative inner body cells surrounded by cap cells, that directs ductal growth throughout the fat pad. The cap cells are highly proliferative with more differentiated cells along the duct that gives rise to luminal and myoepithelial cell layer^60^. Hence the increase in TEBs formation further supports the role of GFs in cellular proliferation and how it governs molecular phenotypes in vivo. Our results are also consistent with the dysregulated branching morphogenesis and reduced TEBs formation as observed in mice mutant for EGF and FGF signaling^61,62^. We have also shown that organoids initially grown in the presence of single GFs can respond to the cocktail of all GFs leading to significantly increased size and ability to form bi-layered epithelial structures. Taken together, our findings strongly suggest the contribution of multiple GFs in mammary organoid maintenance in vitro and their impact on mammary gland regeneration after transplantation in vivo*.*

## Conclusion

We adopt a useful set of experimental tools involving 3D in-vitro culture, whole animal in vivo imaging, ultrasound imaging and ex vivo approaches including tissue clearing and confocal imaging of dissected glands to study mammary gland biology. We anticipate these will open novel avenues to study role of transgenes in mammary gland regeneration in vivo. The recent development of intravital imaging of mouse mammary gland^32^ will be an useful toolbox to trace ductal development over time and study the dynamics of mammary cells grown under different GFs. The current technical advancement of tissue clearing has several advantages and applications over conventional tools. The efficient FGCS-based tissue clearing allowed us to image distinct developmental stage during mammary epithelial morphogenesis, with the presence of terminal end buds (TEBs) in virgin animal and formation of lobulo-alveolar units during pregnancy. The imaging tool used together with tissue clearing that can be applied to whole gland/organoid/tissue will advance our understanding of organ regeneration and development. The optical clearing method can be applied to study regeneration of other mammalian organ system. The ultrasound imaging can also be applied to detect mammary preneoplastic lesions that can be coupled with a fluorescence based whole animal imaging. Future work aimed at different combination of GFs will help to reveal precise culture conditions of mammary organoids and also for studying the role of other genetic regulators that can influence mammary gland morphogenesis. Several of the aforementioned GFs plays important roles, not only during embryonic development but become dysregulated in the adult stage to contribute to cancer pathogenesis. Hence understanding the function of GFs in organoids provides novel insights to cell proliferation, stem cell maintenance, tissue repair and organ regeneration in vivo*.*

## Material and methods

### Mice

Rosa26-tdTomato mice, C57/Bl6 and immunocompromised athymic nude mice [NCr-nu/nu] were maintained in house. Mice expressing a recombined, activated tdTomato allele ubiquitously (Rosa-tdTom-Active) were generated by crossing with a germ-line active Cre transgenic line (PrxCre^63^), followed by breeding to remove the Cre allele in subsequent generations. All animal studies were performed as per the protocols outlined in the Guide for the Care and Use of Laboratory Animals (National Research Council; 2011; National Academies Press; Washington DC, USA) and approved by the NCI-Frederick Animal Care and Use Committee (ASP # 18-471). All animal studies were performed in compliance with ARRIVE (Animal Research: Reporting In Vivo Experiments) guidelines (https://arriveguidelines.org/arrive-guidelines).

### Organoid culture

The inguinal 4th mammary gland (MG) from both sides were dissected from 12 to 16-week-old female virgin mice and minced using a sterilized scalpel. The finely minced tissue was collected in a falcon tube and digested in 1X Collagenase-Hyaluronidase medium (STEMCELL technologies) containing 3000 U/ml Collagenase, 1000 U/ml Hyaluronidase in DMEM for 2 h at 37 °C. The tissue pieces were dissociated every 30 min during the 2-h digestion period using a P1000 tip (10 times). After the digestion, the cell suspension was centrifuged at 400 g for 5 min. The supernatant with overlying liquefied fat layer was discarded and the pellet was resuspended in 1 volume of Hanks buffer and 4 volume of Ammonium chloride solution (STEMCELL technologies) and incubated on ice for 10 min for red blood cell lysis. The solution was centrifuged at 400 g for 5 min and the pellet was further digested with 2–3 ml of 0.25% trypsin EDTA for 5 min and followed by 2 ml of warm 5 U/ml Dispase and 200 µl of 1 mg/ml DNAse I solution for 5 min. The solution was dissociated to single cell suspension and passed through 40 µm sterile filter and checked under a brightfield microscope to check majority of the cells are as single cell. The resulting solution was centrifuged at 350 g for 5 min. The pellet was mixed with Epicult-B complete media containing ROCK inhibitor (Y27632) and 4% growth-factor reduced Matrigel (Corning, Cat # 354230), and seeded in a 96 well or 24 well ultra-low attachment plate (Corning) and grown in suspension culture. 10^4^ live cells per 500 µl of organoid media were plated into each well of a 24 well Ultra-low attachment plate for organoid growth. The organoids were passaged after 7 days when the organoid density is crowded and branching starts.

The following day individual growth factors or a cocktail of growth factors were added to each well. The organoid culture media with different GFs were supplemented after every 4 days for continuous propagation of organoids.

For Matrigel embedding experiments, single cell suspension was mixed with Matrigel at a concentration of 10 cells/µl and 20 µl of cell-Matrigel dome were plated onto each well of a 24 well plate. The plates containing Matrigel-cell mixture dome was incubated in a humidified incubator at 37 °C to solidify for 30 min. Individual wells were filled with organoid basal media containing individual growth factors or with a cocktail of all growth factors. Brightfield images of organoids were imaged using EVOS digital inverted microscope (Invitrogen). The details and final concentration of recombinant proteins used are as follows—**EGF** (50 ng/ml; Cat # AF-100-15), **FGF2** (10 ng/ml; Cat # 100-18B) **FGF10** (10 ng/ml; Cat# AF 100-26). All recombinant proteins were purchased from Peprotech.

### Cell-titer Glo 3D assay

The mammary glands were digested using collagenase- Hyaluronidase (as described above) and single cells were counted using a countess (Invitrogen) and mixed with 4% Matrigel containing Epicult-B complete media containing ROCK inhibitor (Y27632) and 50 µl of cell-Matrigel suspension (200 cells per well) was seeded in a flat-bottom 96 well ultra-low attachment plate (Corning). The following day 50 µl of Epicult-B complete media containing individual or a cocktail of GFs were added to each well and 2% Matrigel concentration was maintained throughout the culture. The Cell-titer Glo 3D Luminescent Cell Viability reagent (Promega, Cat# G9681) was thawed to room temperature before estimation and 100 µl of the reagent was added to each well and thoroughly mixed to lyse 3D organoids and incubated for 30 min at room temperature and protected from light. The plate was then subjected to a Spectramax iD5 (Molecular Devices) microplate reader to read the luminescence. The raw luminescence values were subtracted from the background and relative luminescence was calculated. The luminescence was averaged from three technical replicates and the values were normalized to 1 for organoids grown in all growth factors and accordingly the relative luminescence is calculated for other conditions to estimate the proliferation of organoids. The data were plotted from 2 biological replicates, each containing 3 technical replicates per condition.

### Organoid forming efficiency and size measurement

Single cells isolated from mammary gland digestion were counted using a countess (Invitrogen) and cells were mixed with 4% Matrigel at a concentration of 200 live cells per well of a 96 well ultra low- attachment plate (Corning). The following day different growth factors were added into each well and cultured for 10 days. The growth factors were replenished after 4 days. Each growth factor condition was plated into 6 technical replicates and brightfield images of organoid were taken using Celigo Imaging cytometer (Nexcelom Bioscience). Total number of branched and circular organoids for each condition were counted and percentage was taken to the initial number of cells plated per well. The data is presented by taking average of 6 technical replicates per condition and presented from 2 biological replicates. For measuring the organoid size over a time course, organoids were imaged at day 1, 4, 7 and 10 using EVOS digital inverted microscope (Invitrogen) and diameter was measured using the line tool in Fiji. 30–50 organoids per well were counted for each condition and average diameter was calculated.

### Immunofluorescence and confocal microscopy

Organoids grown in 2% Matrigel suspension were collected into 15 ml falcon tube using P200 tip and briefly washed with 1X PBS and fixed in freshly prepared 4% paraformaldehyde (PFA) made in 1X PBS buffer, for 30 min in room temperature. Organoids embedded in complete Matrigel were incubated with 500 µl of Cell recovery solution (corning) for 1 h to depolymerize Matrigel. The organoids were collected in an Eppendorf tube and gently mixed to ensure Matrigel is completely dissolved and then briefly washed with 1X PBS before adding 4% PFA. Fixative was removed and washed once with 1X PBS. 300 µl of PBS-Tx0.3 (containing 0.3% triton-X) was added to each tube to permeabilize for 30 min and then transferred to a blocking solution (0.5% BSA and PBS-Tx0.3) containing 5% animal serum for 1 h. 50 μl of primary antibody solution (diluted in blocking solution) was added to individual tubes making sure all organoids are covered by the antibody solution and incubated overnight at 4 °C. The following day, the organoids were washed three times with PBS-Tx0.3 each for 30 min. The secondary antibodies were diluted in blocking solution and incubated overnight. The organoids were washed three times with PBS-Tx0.3 each for 30 min and followed by nuclear staining with DAPI for 6 -12 h. The organoids were then mounted on a slide using 80% glycerol or Fructose-Glycerol solution. The antibodies and the corresponding dilution used for immunofluorescence are as follows—**Cytokeratin 8/18** (DSHB, Cat# TROMA-I, 1:250), **Cytokeratin 5** (Biolegend, Cat# 905504, 1:200), **Cytokeratin 14** (Biolegend, Cat# 905301, 1:200), **p63** (Abcam, Clone 4A4, Cat# AB735, 1:40), **ID4** (Biocheck BCH9/82-12, 1:1000), **SMA** (Sigma Aldrich, clone 1A4 Cat # A2547, 1:200), **DsRed2** (Santacruz Cat # SC-101526)**, PR** (Cell Signaling Technology, Cat# 8757T, 1:25), **ERa** (BioRad, Cat # MCA1799T, 1:25) The corresponding secondary antibodies conjugated to Alexa Fluor 488 or Alexa fluor 568 or Alexa Fluor 594 were purchased from Thermo Fisher Scientific (used at 1:1500 dilution).

The slides were imaged using a 20 × objective lens in Zeiss LSM 780 confocal microscope using Zen-Blue software with the appropriate fluorescent lasers. Images were taken as Z-stacks and stitched and then processed as a maximum intensity projection and pseudo-colored using Fiji software (https://fiji.sc/). All measurements and quantifications were done with Fiji-ImageJ (using cell counter plug in) and normalized to the area of each organoid, as described in respective figure legends.

### Mouse surgery and organoid transplantation

Three weeks old immunocompromised athymic nude mice were used for organoid transplantation surgery. The mice were anaesthetized with 2% isofluorane (by inhalation) and Buprenorphine was injected as a pain killer. The skin in the lower belly and near the 4th mammary gland was cleaned with betadine pads and wiped with alcohol. A small incision, 2–3 mm just below the 4th nipple is made and the clearing was accomplished by cauterizing the main blood vessels that supply the mammary fat pad (MFP), then the fat pad and the vessels overlying the lymph node and the area from the nipple to the lymph nodes were removed. 10^4^ cells were dissociated from organoids generated from Rosa26-tdTomato-active mice and suspended in basal organoid media containing 30% growth factor reduced Matrigel. The cell-Matrigel suspension was injected using a TB syringe. The incision was closed with wound clips and tissue glue. Mice were given 3 drops of 0.25% Bupivacaine, at the incision site and placed in a warm place and monitored until they regained consciousness. The wound clips were removed between 7 and 10 days after surgery and set up for mating (if required) at 6 weeks of age to induce proliferation of the mammary glands and to study stages of mammary gland development. All equipment were sterilized before use and all operations were performed under aseptic conditions. The surgery mice were given wet food after the surgery and closely monitored for 1 week.

### Animal imaging: whole animal in vivo fluorescence

Xenogen IVIS spectrum (Perkin Elmer, Waltham, MA) imaging system was used to perform multispectral whole animal in-vivo fluorescence imaging. Mice were anesthetized in the induction chamber with 3% isoflurane (filtered air (0.2 µm filter) at 1 l/min flow rate used as carrier). Mice were then transferred to the imaging table where isoflurane was reduced to 2%. Multispectral fluorescence scanner parameters were Ex: 430 nm (to obtain autofluorescence spectra), 535 nm (to obtain tdTomato spectra); Em 580, 600, 620, 640, 660, 680 nm; with an autoexposure (1–60 s) at F-stop of 1. Image analysis (Living Image, ver 4.3.1, PerkinElmer) and spectral unmixing (to correct for autofluorescence) was performed in accordance with the manufacturer’s specifications. The left (control) and right (injected) side of the mice were imaged. Radiant efficiency scale in the whole animal fluorescence is expressed as (p/sec/cm^2^/sr)/(µW/cm^2^).

### Ultrasound imaging

Mammary fat pad was imaged using the Vevo 2100 (VisualSonics, Toronto, Canada) with a MS-550S 40 MHz transducer (40 µm axial image resolution). The mid-section of the mammary fat pad was lined up using 2D (single slice) B-mode and then a 3D image was acquired using a 0.15 mm step size.

### Tissue clearing and whole gland imaging

Four weeks post transplantation, the mammary glands were dissected out from the mice and briefly fixed in 4% PFA (without methanol) for 30 min at room temperature. The glands were briefly washed in 1X PBS for another 30 min and then incubated in Fructose-Glycerol clearing solution (FGCS) containing 60% (vol/vol) glycerol and 2.5 M fructose (as described in^36^). Tissue clearing was performed for 24 h (Virgin mice) or for 3–7 days (Pregnant mice) depending on the stage of mammary gland development. The glands were mounted using a cover slip and FGCS and sealed by nail polish before imaging. Pregnant glands were incubated for another 48–72 h (due to thickness of the tissue) before mounting. The glands were imaged using Zeiss LSM 780 confocal microscope with tile scanning and Z-stack imaging. The whole image was stitched and processed for maximum intensity projection using ZEN blue software.

### Statistical analysis

Experiments were repeated at least twice and statistical details for each experiment can be found in the figures and the legends. Statistical analysis was performed using Graphpad Prism. V9. Statistical significance is calculated by Fisher’s Exact test (Two-tailed), Student’s t test and one-way ANOVA for multiple comparison test was used. Results are represented as mean +/− SD as indicated in the figures and *p *values are indicated with *p < 0.01, **p < 0.001, ***p < 0.0001, ns = not significant.

## Supplementary Information

Supplementary Figures.

## Data Availability

All data generated or analyzed during this study are included in the manuscript and supporting files.

## Abbreviations

tdTomato: Tandem dimer Tomato
3D: Three dimensional
3DISCO: 3D imaging of solvent-cleared organs
BLI: Bioluminescence imaging
BSA: Bovine serum albumin
CUBIC: Clear, unobstructed brain or body imaging cocktails and computational analysis
CLARITY: Cleared lipid-extracted acryl-hybridized rigid immunostaining/in situ hybridization-compatible tissue hydrogel
EDTA: Ethylenediaminetetraacetic acid
eGFP: Enhanced green fluorescent protein
EGF: Epidermal growth factor
FGF: Fibroblast growth factor
FGCS: Fructose and glycerol clearing solution
GF: Growth factor
MaSCs: Mammary stem cells
PFA: Paraformaldehyde
ROCK: Rho-associated kinase
SHANEL: The small micelle-improved human organ ANtibody efficient labeling
TEB: Terminal end bud
TF: Transcription factor
