# Supplementary material for: Growth factor dependency in mammary organoids regulates ductal morphogenesis during organ regeneration
Source: Sci Rep. 2022 May 3;12:7200. doi: 10.1038/s41598-022-11224-6 (PMC9065107; doi:10.1038/s41598-022-11224-6)
Supplement: Supplementary file 1 — Supplementary Figures. [file 41598_2022_11224_MOESM1_ESM.docx]

 **Supplementary information**

**Growth factor dependency in mammary organoids regulates ductal morphogenesis during organ regeneration**

Sounak Sahu^1^, Mary E Albaugh^1,2^, Betty K. Martin^1,2^, Nimit L. Patel^2,3^, Lisa Riffle^2,3^, Susan Mackem^4^, Joseph D. Kalen^2,3^, Shyam K. Sharan^1,5*^

**
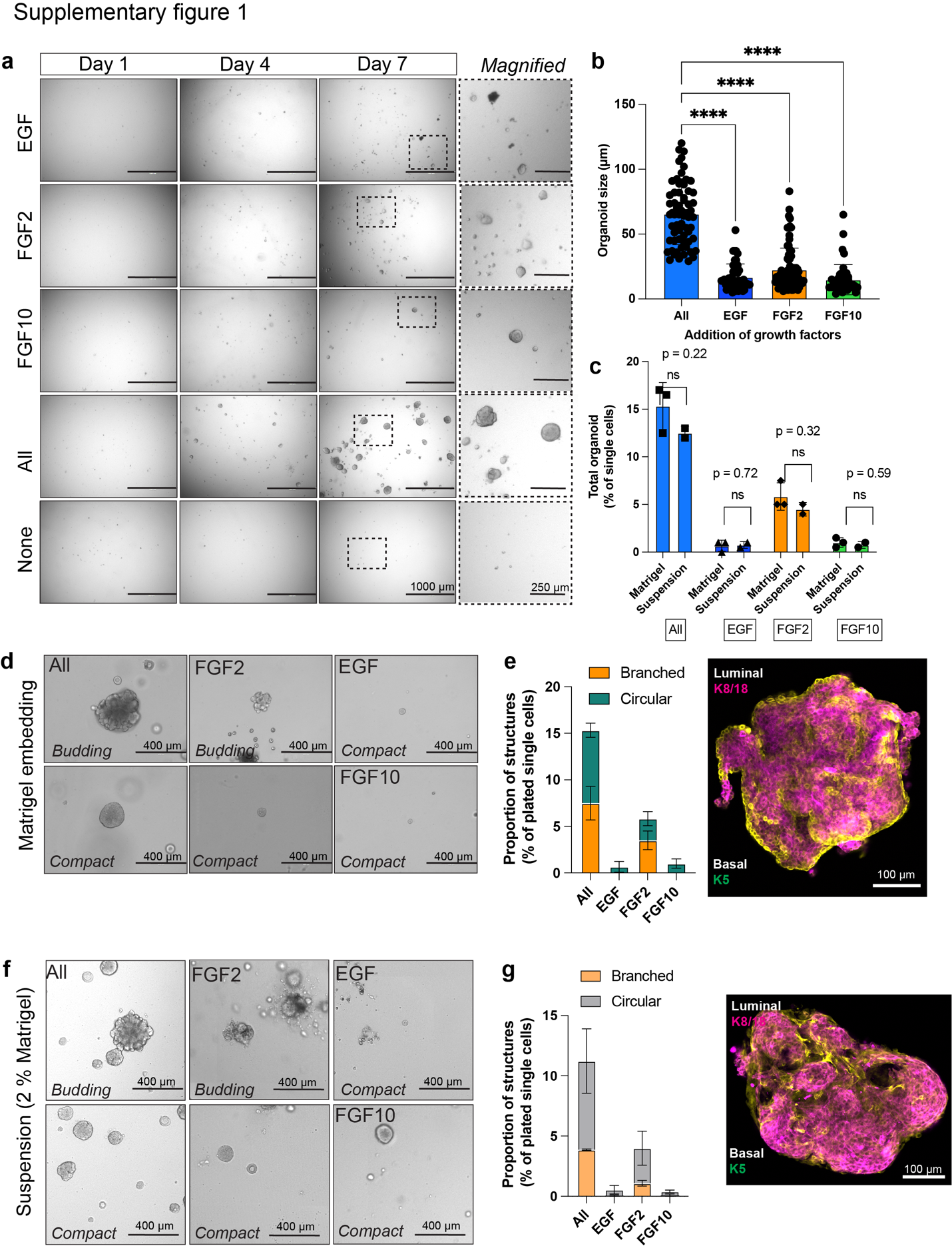
**

**Supplementary figure 1: Role of growth factors in mammary organoid generation.**

**(a)** Time course (day 1 to day 10) showing the development of organoids from single isolated mammary epithelial cells cultured *in vitro* in the presence of individual GFs (EGF or FGF2 or FGF10) or in a combination of all GFs. Organoids are not formed in the absence of any growth factors to the organoid media. **(b)** Organoid size was measured from individual organoids grown in the presence of GFs. Each dot represents size of individual organoids. (n = 65 for all, n = 50 for EGF, n = 79 FGF2, n = 45 for FGF10)**.** One-way ANOVA was used for calculating statistical significance (**** p<0.0001). **(c)** Total organoid efficiency (branched and compact) formed in suspension culture (containing 2% Matrigel) and in complete Matrigel embedding methods. Percentage is calculated based on total number of cells plated into each well. Each dot represents percentage of organoids formed, n = 3 technical replicates for Matrigel embedding methods and n = 2 independent experiments for suspension culture. Students t-test was used to calculate significance between suspension and complete Matrigel embedding methods. Brightfield images of organoids with branched structures and compact/circular structures formed under different GF condition and grown in complete Matrigel embedding method **(d)** and in suspension method **(f).** **(**Quantification of total number of branched and compact structures grown in presence of single GFs after embedded in complete Matrigel **(e)** or suspension culture containing 2% Matrigel **(g).** Representative image showing the presence of luminal K8/18^+^ cells and Basal K5^+^ cells in organoids. No branched organoids were observed when cultured in the presence of EGF or FGF10 alone.

**
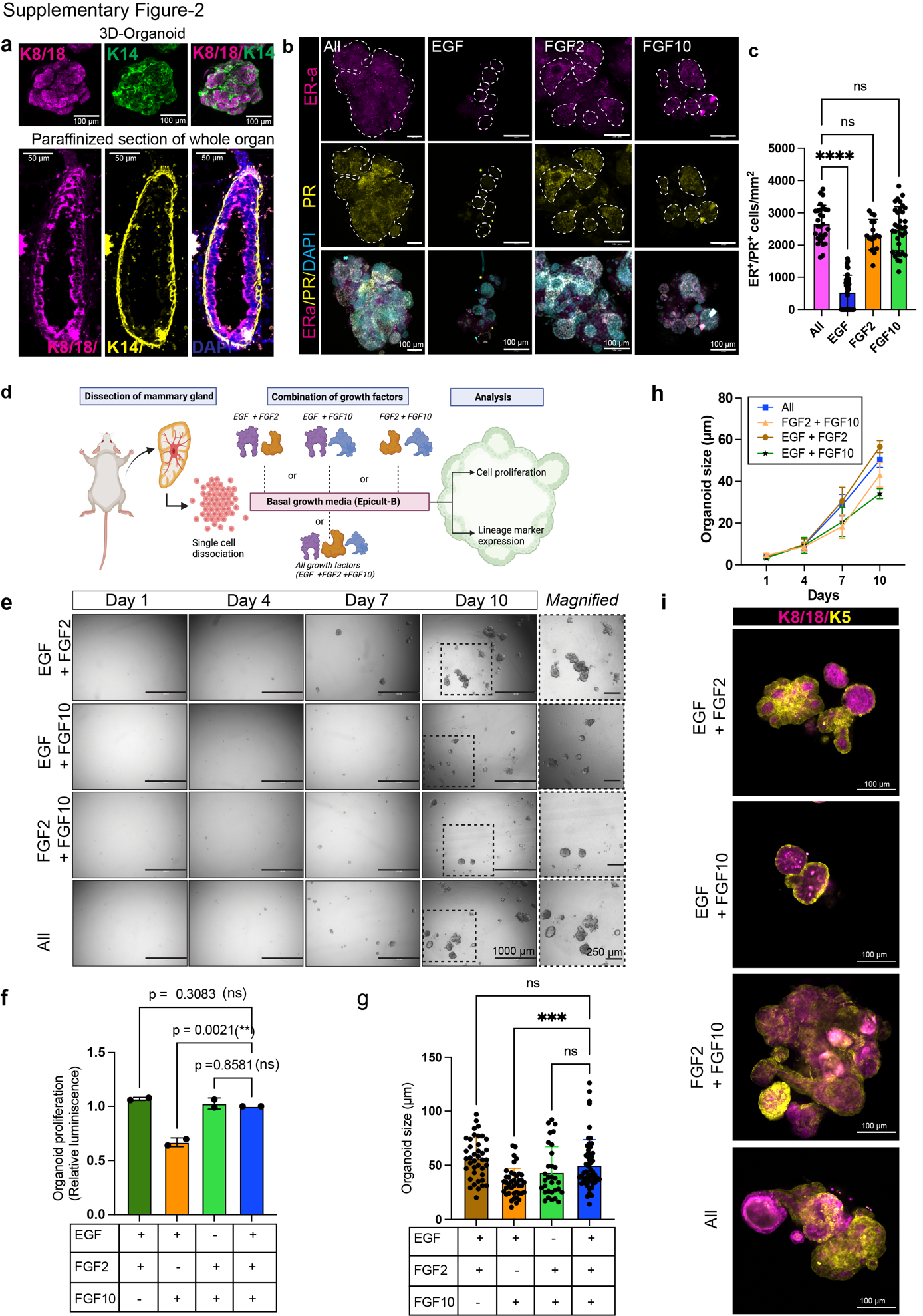
**

**Supplementary figure 2: Effect of individual growth factors in maintaining hormone responsive cells in mammary organoids and role of combination of GFs in organoid maintenance.**

**(a)** Immunostaining showing the expression of basal K5 and luminal K8/18 markers in *in vitro* cultured 3D mammary organoids (scale bar = 100 µm) and paraffinized section of adult mammary gland (scale bar = 50µm). Organoids recapitulates its lineage markers expression like the adult mammary gland. **(b)** Immunostaining showing the presence of Estrogen receptor (ER) and Progesterone receptor (PR) in organoids cultured under different growth factors. Mammary organoids grown in EGF only have significantly reduced ER and PR positive cells (Scale bar = 100 µm) **(c)** Quantification of ER^+^ and PR^+^ cells/mm^2^ in individual organoids. (n = 28 for all, n = 38 for EGF, n = 16 for FGF2, n = 34 for FGF10) (One way ANOVA, ****p<0.001, ns = not significant, p= 0.242 for FGF2, p =0.6920 for FGF10). **(d)** Experimental schematic showing mammary glands were surgically dissected and dissociated into single cells and were cultured in 3D in the presence of combination of GFs (EGF+FGF2, EGF+FGF10, and FGF2+FGF10, and in combination of all GFs (EGF+FGF2+FGF10). The mammary organoids were further analyzed for cell proliferation and expression of mammary lineage markers **(e)** Representative images showing the size of organoids cultured in a combination of GFs over a time course from Day 1, 4, 7 and 10 (Scale bar = 1000 µm) **(f)** Organoid proliferation at day 10 is maintained when cultured in combination of GFs except in the absence of FGF2 in the organoid media. Results are expressed as mean ± SD where each dot represent a biological replicate (n = 2 biological replicates containing 3 technical replicates each). One way ANOVA was used for computing statistical significance in comparison to the cocktail of all GFs (**p<0.01, ns = not significant, p = 0.3083 for EGF + FGF2, p = 0.85 for FGF2 + FGF10). **(g)** Quantification of organoid size at day 10 for all combination of GFs show reduced organoid size in the absence of FGF2. One way ANOVA was used for computing statistical significance in comparison to cocktail of all GFs (***p<0.001, ns = not significant, p = 0.44 for EGF+FGF2, p = 0.46 for FGF2+FGF10). **(h)** Quantification showing the growth of organoids over a time course starting at day 1 to day 10. (n>50 organoids were counted per time-point per condition). **(i)** Immunofluorescent staining showing the maintenance of Cytokeratin 8/18 (Magenta, luminal epithelial cell markers) and Cytokeratin 5 (Yellow, basal epithelial cell marker) in organoids grown under different combination of GFs. (Scale bar = 100 µm).

**
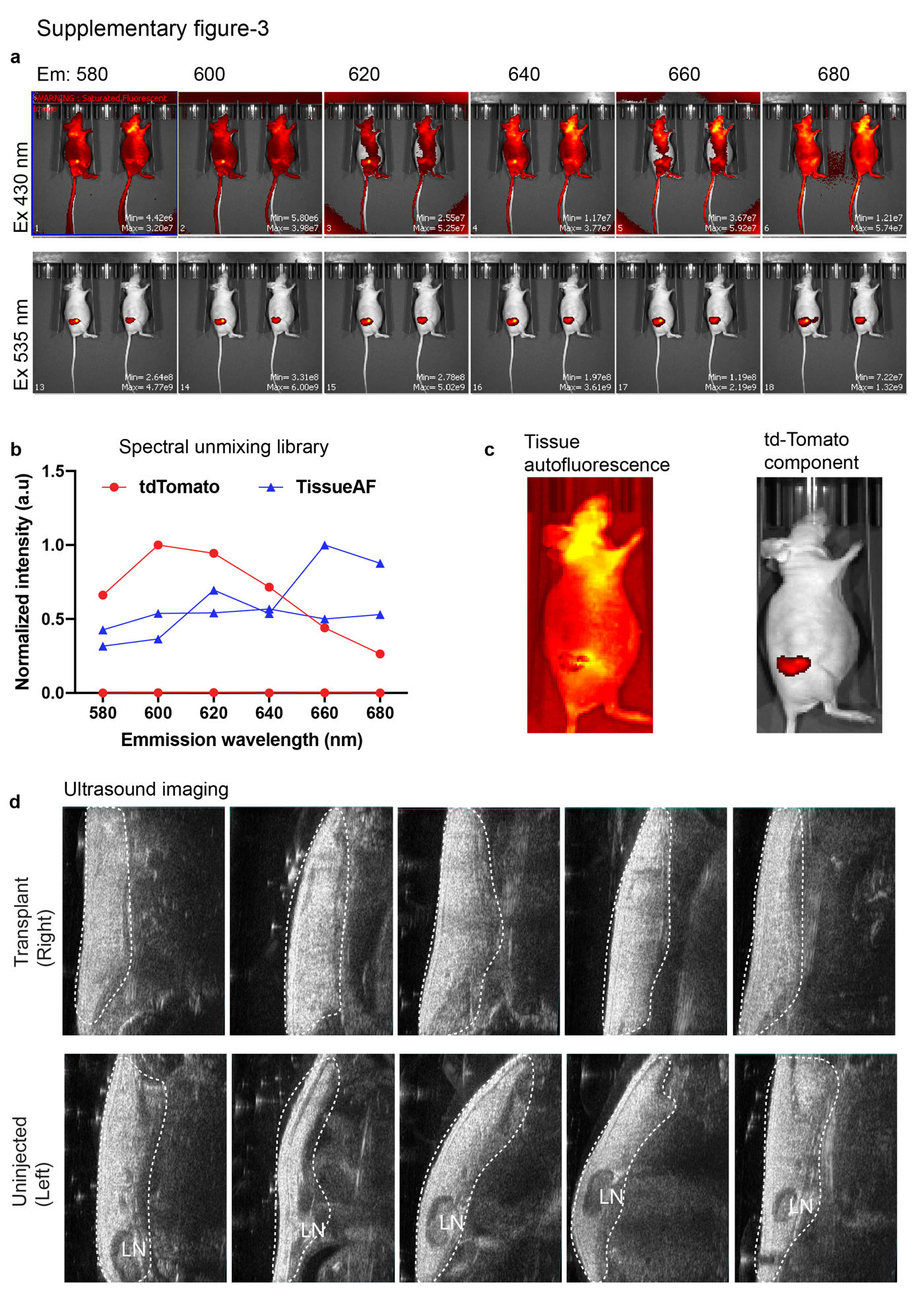
**

**Supplementary figure 3: Optical un-mixing to analyze whole animal tdTomato fluorescence imaging**

**(a)** Raw images of whole animal fluorescence showing excitation at 430 nm to assess skin autofluorescence and 535 nm to excite tdTomato, and the corresponding emission acquired at 580, 600, 620, 640, 660 and 680 nm **(b)** Spectral library obtained from the raw images which was utilized to deconvolve the tdTomato fluorescence signals from the skin autofluorescence. The two values to tdTomato and Tissue autofluorescence (tissueAF) corresponds to the left and right side. The un-injected control side shows negligible fluorescence for tdTomato. **(c)** Representative images showing result of the unmixing process. Skin autofluorescence is evident throughout the mouse body in the “tissue autofluorescence” channel. Presence of a localized signal at the injection site in the tdTomato channel confirms the presence of tdTomato^+^ mammary organoids. **(d)** Ultrasound Images of individual mice (n = 5 mice) showing the mammary fat pad from the right side (tdTomato expressing organoid transplant after fat pad clearing) and from left side (Un-injected control). Lymph node (LN) is visible in the un-injected control side where mammary fat pad was not cleared.

**
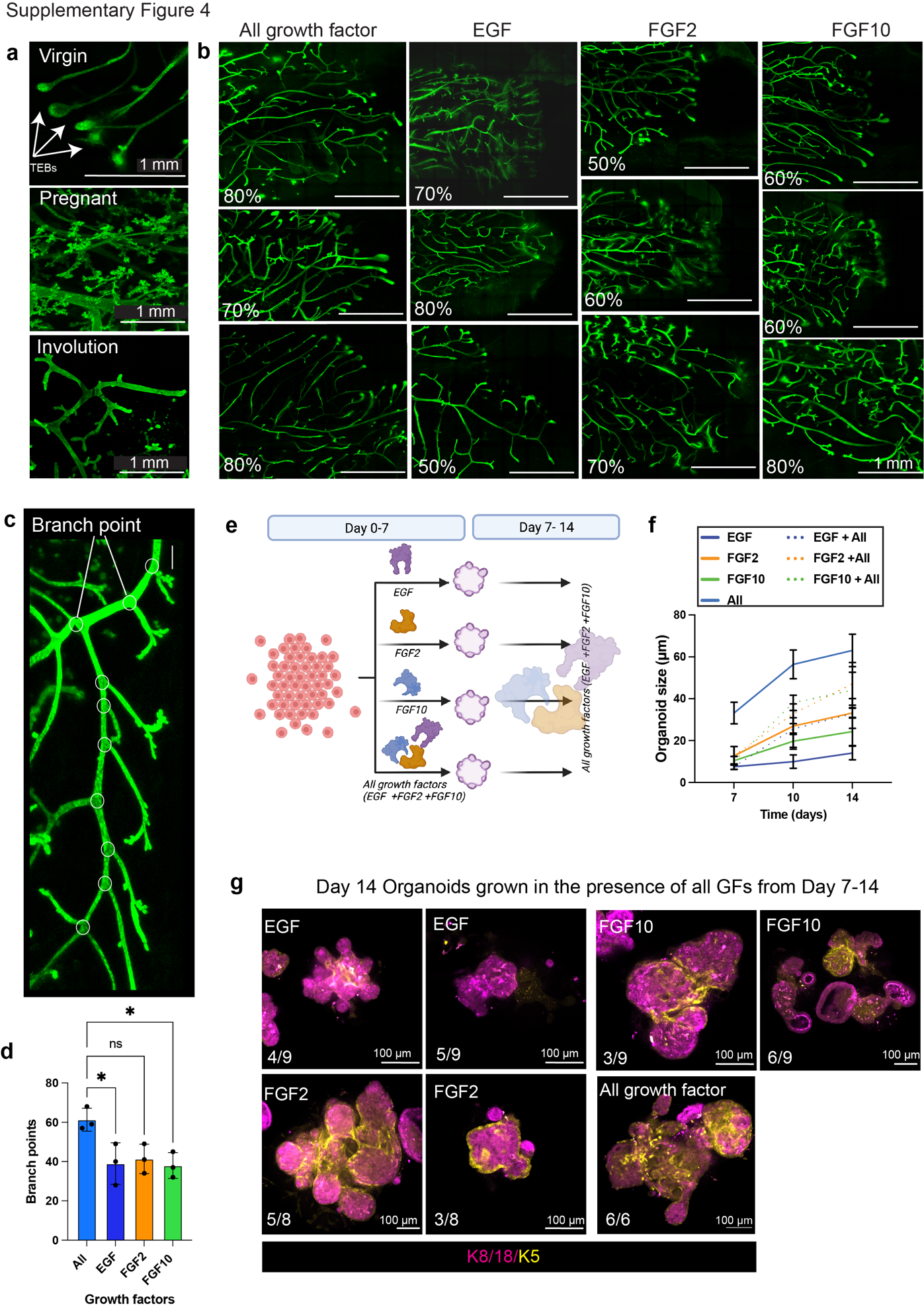
**

**Supplementary figure 4: *Ex vivo* imaging allows study of mammary developmental stages and reveals the role of growth factors in mammary gland reconstitution *in vivo***

**(a)** *Ex vivo* confocal imaging on dissected mammary gland for tdTomato fluorescence recapitulates distinct developmental stages of mammary gland development. The formation of terminal end buds (arrow, TEBs) in virgin animals at 4 weeks post transplantation, development of extensive branching of mammary ducts and lobulo-alveolar units during pregnancy and involution of the ducts post-lactation. (Scale bar = 1 mm) **(b)** tdTomato endogenous fluorescence imaging from mammary fat pads dissected from individual mice transplanted with organoids grown in the presence of different growth factors (n = 3 mice per condition). The percentage denotes the fat pad filling per gland. **(c)** Representative image of the ductal branch points quantified from the mammary fat pads **(d)** Quantification of branch points per gland (n = 3 mice per condition, One way ANOVA, *p<0.05, ns = not significant). **(e)** Experimental schematic to investigate the effect on organoids when cocktail of all GFs was substituted to organoids grown in single GFs. Single cells isolated from digested mammary gland were cultured in the presence of single GFs for the initial 7 days. Cocktail of GFs were added to the organoids at day 7 and cultured for another 7 days and mammary organoid size is counted over a time course and fixed to check the expression of lineage markers. **(f)** Increase in organoid size when cocktail of GFs was added to the organoids initially grown in the presence of individual GFs. Data is represented as average of 3 technical replicates each from 2 independent experiments. Single GFs were continued as a control for these experiments represented as straight lines. The dashed lines denote the organoid size when all GFs were added. **(g)** Representative images of immunofluorescent staining showing the presence of K5^+^ and K8/18^+^ cells in the organoids that were grown in the presence of all GFs from Day 7-14. The numbers denotes the number of organoids used for staining per condition. Organoids grown in all GFs were used as a control to represent the presence of bilayered epithelial cell types.
